# Unexpected trophic diversity in the endemic fish *Orestias chungarensis* in a high‐altitude freshwater ecosystem, Lake Chungará (4520 m), northern Chile

**DOI:** 10.1111/jfb.70081

**Published:** 2025-05-21

**Authors:** Karina González, Daniel Gomez‐Uchida, Chris Harrod

**Affiliations:** ^1^ Doctorado en Ciencias Aplicadas mención Sistemas Acuáticos Facultad de Ciencias del Mar y Recursos Biológicos, Universidad de Antofagasta Antofagasta Chile; ^2^ Fish and Stable Isotope Ecology Laboratory Instituto de Ciencias Naturales Alexander von Humboldt, Universidad de Antofagasta Antofagasta Chile; ^3^ Millenium Nucleus of Austral Invasive Salmonids (INVASAL) Concepción Chile; ^4^ Genomics in Ecology, Evolution and Conservation Laboratory, Departamento de Zoología Facultad de Ciencias Naturales y Oceanográficas, Casilla 160‐C, Universidad de Concepción Concepción Chile; ^5^ Universidad de Antofagasta Stable Isotope Facility (UASIF), Instituto Antofagasta, Universidad de Antofagasta Antofagasta Chile; ^6^ Scottish Centre for Ecology and the Natural Environment, School of Biodiversity One Health and Veterinary Medicine, University of Glasgow Glasgow UK

**Keywords:** Andean fish, ecotype, stable isotopes, stomach contents analysis, trophic diversity

## Abstract

*Orestias chungarensis* Vila & Pinto, 1986 is a small‐bodied (max fork length = 120 mm) cyprinodontiform fish with a very restricted global distribution. The species is limited to a single, small (283 km^2^), high‐altitude (4520 m) catchment located in the Altiplano of northern Chile. Until the late 20th century, *O. chungarensis* was the only fish species inhabiting both Lake Chungará and its main afferent river, the River Chungará. The introduction of rainbow trout [*Oncorhynchus mykiss* (Walbaum, 1792)] at this time raised concerns for the long‐term conservation of *Orestias*. By 2017, *O*. *chungarensis* were no longer present in the River Chungará but remain relatively numerous in Lake Chungará. Although *O. chungarensis* are of elevated conservation concern, little is known regarding their ecology, and the few studies conducted have relied on individuals captured from shallow littoral habitats. Here, we captured *O. chungarensis* from different lake habitats and analysed multi‐tissue stable isotopes (δ^13^C, δ^15^N, δ^34^S) and stomach contents to characterise their trophic ecology. We also used geometric morphometrics to analyse any putative habitat‐associated variation in body shape. *O. chungarensis* showed very wide variation in their stable isotope values (range: δ^13^C = −15.1 to −8.0‰; δ^15^N = 8.9–14.1‰; δ^34^S = −10.5–1.7‰). A *k*‐means cluster analysis indicated that individuals could be best classified into two groups in stable isotope space. A discriminant function analysis supported the separation of the sampled population into two groups (jack‐knifed classification success = 98%). Individuals belonging to either a putative littoral group (^13^C‐enriched, ^15^N‐depleted and ^34^S‐depleted) or a group associated with pelagic‐derived materials (^13^C‐depleted, ^15^N‐enriched, ^34^S‐enriched), which likely fed offshore or in deeper waters. Stomach contents results showed that *O. chungarensis* from the two putative groups had consumed similar prey prior to capture, feeding mainly on benthic macroinvertebrates (amphipods, chironomid larvae and pupae and gastropods). Mixing models analysis showed a broadly similar diet between groups, but the scale of contribution to the assimilated diet differed between groups. Comparisons of stable isotope niche size and overlap showed limited niche overlap, providing more evidence for differential foraging patterns. The dichotomy between the results from stable isotope and stomach content analysis suggests that *O. chungarensis* individuals forage on taxonomically similar diets, but their prey are fuelled from materials derived from different lake habitats (littoral and open‐water). Given the remarkable plasticity found in the genus, our results could reflect the existence of a previously unrecognised ecotype.

## 1 INTRODUCTION

The high conservation value associated with tropical high‐altitude Andean lakes is based on a number of factors as refugia for rare species and communities, reservoir of water, sentinels of environmental change (Jacobsen & Dangles, 2017), foremost of which is their biodiversity (Myers et al., 2000). These are poorly understood ecosystems, with limited information available on their basic ecological processes (Jacobsen & Dangles, 2017). High Andean lakes characteristically have reduced species diversity, and prior to human introductions, these were either naturally fish‐free or supported endemic fish communities (Jacobsen & Dangles, 2017). A key fish genus inhabiting Andean tropical lakes is *Orestias* [family: Orestiidae; order Cyprinodontiformes (Morales et al., 2024)]. This genus is recognised for its high endemism and adaptation to the abiotically extreme high‐altitude systems of the Andes (Vila et al., 2007). The taxonomy, phylogenetics and phylogeography of the genus have been studied in marked detail (Parenti, 1984a, 1984b; Takahashi & Moreno, 2015; Vila et al., 2013). Conversely, far less effort has been expended on understanding its ecology (López et al., 2023; Vila et al., 2007). The genus *Orestias* inhabits a range of inland waters across the Altiplano (Andean high plateau) of Bolivia, Chile and Perú (Parenti, 1984b). Many such systems support a single allopatric *Orestias* population, which are recognised as distinct species (Vila et al., 2013). However, these *Orestias* spp. have common and strong habitat associations with macrophytes where they shelter, feed and reproduce (Pinto & Vila, 1987; Riveros et al., 2012; Villwock & Sienknecht, 1996). However, lakes include a variety of habitats associated with different potential prey that can be used by fishes, for example, zooplankton in pelagic habitats and benthic macroinvertebrates in deeper habitats away from the littoral (Robinson & Wilson, 1994; Schluter, 1996; Smith & Skúlason, 1996). Under suitable conditions, *Orestias* can undergo adaptive radiation, such as in the large (8372 km^2^) high‐altitude (3812 m asl) Lake Titicaca (Bolivia/Peru) where up to 15 *Orestias* species have evolved with distinct morphology (Maldonado et al., 2009), trophic ecology (Dejoux & Iltis, 1992) and stable isotope values (Monroy et al., 2014), which are associated with different lake habitats.

Lake Chungará (4520 m asl) is located in the Altiplano of Northern Chile, ca. 190 km to the south of Lake Titicaca. In contrast to Titicaca, until recently, Lake Chungará only supported a single native fish species, *Orestias chungarensis* (Vila & Pinto, 1986). Originally described from the lake, *O. chungarensis* was also present in large numbers in the afferent River Chungará in 2007–2008 (Harrod, unpublished).

At an undefined date in the late 20th century, rainbow trout *Oncorhynchus mykiss* (Walbaum, 1792) were introduced to the Chungará catchment, where they rapidly became established and now dominate both lake and river habitats (González et al., 2024). By 2017, *O*. *chungarensis* were no longer present in the River Chungará (authors’ personal observation), and the loss is likely associated with the introduction of rainbow trout which consume *Orestias* (González et al., 2024). Restricted to a single isolated lake and subject to an array of threats, *O. chungarensis* is considered as critically endangered by the International Union for Conservation of Nature (IUCN) (https://www.iucnredlist.org/species/15492/176559909). However, little is known about its ecology (Guerrero et al., 2015; López et al., 2023), and such information (e.g., diet, population structure) is urgently required to permit the conservation of *O. chungarensis*.

The few studies detailing the trophic ecology of *O. chungarensis* have relied on individuals captured from the shallow littoral (Guerrero et al., 2015; Vila & Pinto, 1986); they also assumed that the lake supports a single population of *Orestias* using stomach content analysis, an approach that provides valuable data at a high taxonomic resolution. However, dietary information is limited to which prey are consumed to the days or hours prior to capture rather than what materials are assimilated and used to build tissues (Harrod & Stallings, 2022; Majdi et al., 2018; Nielsen et al., 2017). Stomach content analysis may fail to identify habitat‐associated differences in consumer diet if prey are ubiquitous and inhabit different habitats across environmental gradients (e.g., lake depth). An alternative approach is to combine stomach content data with biochemical measures, such as stable isotope analysis (SIA), as dietary tracers (Harrod & Stallings, 2022; Majdi et al., 2018; Nielsen et al., 2017). SIA provides information on assimilated diet, and depending on the tissue sampled, it provides information on assimilated prey over longer temporal scales (weeks – months) than that of analyses of stomach contents (Thomas & Crowther, 2015). Isotopic differences between habitats mean that stable isotope values can provide information on where consumers have foraged (Fry, 2002; Harrod et al., 2010). Consumer stable isotope data can also be used to derive measures of ecological interest, including trophic position, individual specialisation and isotopic niche size (Araújo et al., 2007; Harrod et al., 2010, 2005; Matthews & Mazumder, 2004).

During a recent study of rainbow trout diet in the Chungará catchment (González et al., 2024), *O. chungarensis* were captured across a range of habitats. González et al. (2024) showed that *O. chungarensis* displayed marked inter‐individual variation in carbon, nitrogen and sulphur stable isotope values, with individuals falling into at least two distinct isotopic groups. Given the size of Lake Chungará and the capacity of *Orestias* to adapt to potentially unoccupied niches elsewhere (e.g., Lake Titicaca), it seems probable that there is an unrecognised trophic diversity in the Lake Chungará population. Moreover, *O. chungarensis* is included in the *Orestias agassii* complex (Parenti, 1984a), a group that includes species inhabiting benthic, pelagic and littoral habitats across the Altiplano (Vila et al., 2007).

Morphology provides reliable information regarding the ecological niche of fish both at the species and individual levels (Winemiller, 1991), and body shape is a reliable indicator of habitat preferences in fishes (Keast & Webb, 1966). The characterisation of shape variation using geometric morphometrics (Adams et al., 2004; Parsons et al., 2003) has been useful in identifying different groups of consumers and estimating phenotype‐environment correlations (Harrod et al., 2010; Zimmerman et al., 2009).

To evaluate the possible existence of trophic variation in this isolated population, we used multiple approaches to examine how habitat use and trophic ecology varied in *O. chungarensis*. We used stomach content analysis to characterise diet in the short term (hours prior to capture) and SIA (δ^13^C, δ^15^N, δ^34^S) to examine their assimilated diet in longer periods, that is, liver (∽ 6 weeks) and muscle (∽ 6 months). Also, we use geometric morphometrics to examine possible variation in body shape of *O. chungarensis*. We assume that any variation recorded by these measures will scale with the existence of currently unrecognised ecological diversity within the population.

## 2 MATERIALS AND METHODS

### 2.1 Study area

**FIGURE 1:**
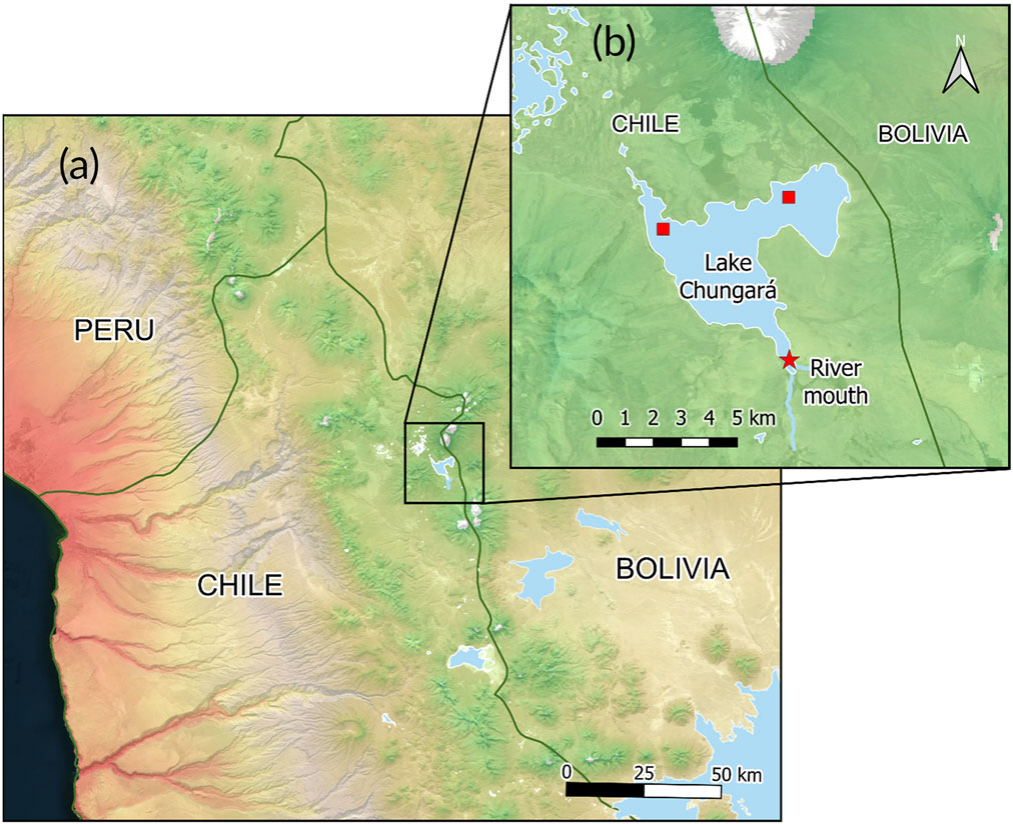
(a) Location of Lake Chungará in the Altiplano, (b) showing *Orestias chungarensis* sampling sites (red squares) and the location of the river mouth of the River Chungará.

Lake Chungará (18°15′ S, 69°09′ W, 4520 m asl, Figure 1) is located in the Lauca National Park, which was declared a UNESCO Biosphere Reserve in 1981 (https://www.unesco.org/en/mab/lauca). It is situated close to the international borders between Chile, Bolivia and Peru. Lake Chungará is a polymictic system of tectonic‐volcanic origin, with a surface area of 22.5 km^2^, a water volume of 426 × 106 m^3^ and a maximum depth of 34 m (Mühlhauser et al., 1995). Its main tributary is the River Chungará, a small river (length < 10 km, mean width = 1.8 m, mean depth = 0.4 m) that drains the area to the south and southeast of the lake.

The littoral zone of Lake Chungará supports a patchy but often dense curtain of macrophytes (e.g., *Myriophillum elatinoides*, *Potamogeton filifolius*), as well as microbial colonies that also contribute to primary productivity (Dorador et al., 2003). Macrophyte patches are an important habitat for aquatic birds (Rundel & Palma, 2000), as well as for the littoral macroinvertebrate community, which is largely composed of insects in different stages of development, amphipods, gastropods and bivalves. The phytoplankton community of the lake is species poor and includes diatoms and chlorophyceans that dominate during the cold and warm seasons, respectively (Dorador et al., 2003). The zooplankton community is dominated by calanoid copepods (Andrew et al., 1989) and cladocerans (Mühlhauser et al., 1995).

### 2.2 Sample collection

#### 2.2.1 Fish

*O. chungarensis* (*n* = 42) were captured from lake using standard CEN (European Standard, 2005) benthic multipanel nets (2.5 × 1.5 m panels of 29, 35, 5, 15.5, 24, 12.5, 8, 55, 10, 6.25 and 19.5 mm mesh) and fyke nets. Multipanel and fyke nets were set at depths between 2.5 and 10 m, respectively, in the later afternoon, fished overnight and lifted/checked in the following morning.

#### 2.2.2 Putative prey

We sampled a range of putative *O. chungarensis* prey from the lake. Zooplankton were collected using vertical hauls of 200 μm nets (opening area: 0.125 m^2^, length: 100 cm) from three sites, and filtered samples were placed in 500 mL bottles and preserved on ice for 8 h. Littoral and sub‐littoral macroinvertebrates were collected using a van Veen grab (volume: 400 cm^2^, dimensions: 550 × 340 × 220 mm) from depths of 2, 5, 10 and 15 m. Organisms were removed from the sediments, placed in labelled plastic bags and stored on ice. Littoral prey were also obtained from stomach contents of littoral (river mouth) rainbow trout (see González et al., 2024) following Grey et al. (2002).

### 2.3 Laboratory work

*O. chungarensis* were measured for length (fork length: ± 1 mm) and mass (blotted wet mass ± 0.1 g). Their stomachs were dissected out and frozen at −20°C. Samples of liver and white dorsal muscle were taken from each individual for SIA. These tissues were chosen to reflect different turnover rates, which allow them to reflect the assimilation of food during different temporal scales (Thomas & Crowther, 2015). Although we do not have empirical turnover data from *Orestias* spp., we assume that liver tissues will reflect the time‐averaged assimilated diet over approximately 6 weeks and muscle tissue of approximately 6 months (Thomas & Crowther, 2015). As such, by combining stomach content and stable isotope data, we can estimate information on the diet of *O. chungarensis* on a temporal scale, varying from days (stomach contents) to weeks (liver tissue SIA) and many months (muscle tissue SIA) near to a year temporal scale. Tissues were placed into 1.5 mL microcentrifuge vials and then frozen at −20°C. Zooplankton samples were concentrated by filtering them on pre‐combusted GF/F filters (500°C for 4 h); the filters were then preserved at −20°C.

Macroinvertebrate samples were washed with distilled water and preserved at −20°C. These were subsequently defrosted and then identified to the lowest taxonomic level, using a dissection microscope and taxonomic keys (Dejoux & Iltis, 1992; Domínguez & Fernández, 2009; González, 2003) before preparation for SIA.

Sample collection was conducted under permission from the relevant statutory body (SUBPESCA R.Ex.No. 348, February 2016) and covered under the approval of the Universidad de Concepción ethic, bioethics, and biosecurity committee (CEBB permit number: 1337).

### 2.4 Analytical procedures

#### 2.4.1 Determination of stable isotope ratios

*O. chungarensis* muscle and liver tissues, as well as samples of putative prey, were dehydrated for 48 h in a benchtop freeze dryer (LABCONCO FreeZone Plus Cascade, Kansas City, MO, USA). Once dry, samples were stored in 1.5 mL microcentrifuge tubes contained in sealed plastic bags under low humidity conditions for 12 h. Samples were then homogenised (Pérez et al., 2022) in 2 mL screw cap polypropylene tubes (Biologix, Shandong, China) with two 3.2 mm stainless steel beads for 30 s at 3500 oscillations/min using a bead‐beater (Mini‐Beadbeater, Bio Spec Products Inc., Bartlesville, OK, USA). After grinding, approximately 1.5 mg of homogenised tissue was weighed into 8 × 5 mm pressed standard weight tin capsules using a high precision (repeatability = 0.0008 mg) microbalance (model XS 3DU, Mettler Toledo, Greifensee, Switzerland).

Elemental percentages for carbon, nitrogen, sulphur and stable isotope ratios (δ^13^C, δ^15^N and δ^34^S) were measured using a Pyrocube elemental analyser (Elementar, Langenselbold, Germany) linked to a visION continuous‐flow isotope ratio mass spectrometer (Elementar, Langenselbold, Germany) at the Universidad de Antofagasta Stable Isotope Facility (UASIF), Chile. Stable isotope ratios are expressed using δ notation and are reported as per mil (‰) relative to Vienna Pee Dee Belemnite for carbon, air for nitrogen and Vienna Canyon Diablo Troilite for sulphur.

Several international standards were used in each batch to provide a multi‐point calibration using the ionOS software package version 4.1.005 (Elementar, Langenselbold, Germany). Certified reference materials USGS40 and USGS41a were used for carbon and nitrogen and IAEA‐SO‐5, IAEA‐SO‐6 and IAEA‐S2 for sulphur. Repeated analysis of standards showed analytical errors [± 1 standard deviation (SD)] of ±0.04‰ for δ^13^C, ± 0.06‰ for δ^15^N and ± 0.60‰ for δ^34^S. We used two calibration standards: (a) sulfonamide (Elementar, Germany) and (b) an in‐house standard (rainbow trout dorsal muscle) to correct for instrument drift.

Fish, muscle and liver tissues commonly contain lipids that interfere with the estimation of δ^13^C values and may have a non‐dietary origin (DeNiro & Epstein, 1977). As such, we estimated lipid‐free δ^13^C values for *O. chungarensis* using arithmetical corrections based on sample C : N for muscle (Kiljunen et al., 2006) and liver (Logan et al., 2008). We did not correct putative prey δ^13^C based on the assumption that *O. chungarensis* likely assimilate the entire biochemical package provided by prey (lipids, proteins and carbohydrates) – see Kiljunen et al. (2006) for a discussion.

#### 2.4.2 Diet: Stomach contents

*O. chungarensis* stomachs were opened, and their contents placed into Petri dishes. Prey taxa were then identified to the lowest taxonomic level using a dissection microscope and identification keys (see above for details). We estimated the relative importance of different prey taxa to the diet of each *Orestias* using two common approaches (Harrod & Stallings, 2022). Frequency of occurrence (%Fi) provides information on the proportion of all individuals with stomach contents that contained a given taxon and can be used as a qualitative indicator (presence/absence) to which taxa are important in the diet. To estimate the relative contribution (%Pi), which provides information on the proportional contribution of food items to the stomach volume, calculated by applying a semi‐quantitative modified points method (Hyslop, 1980; Swynnerton & Worthington, 1940) (Equation 1).

(1) $$\%P_{i}=\frac{P_{i}}{P_{t}}\times 100\;i$$

where $\%P_{i}$ is the percentage contribution of item i, $P_{i}$ is the value of points allotted to the item i, and $P_{t}$ is the number of points allotted to the stomach.

### 2.5 Shape analysis

**FIGURE 2:**
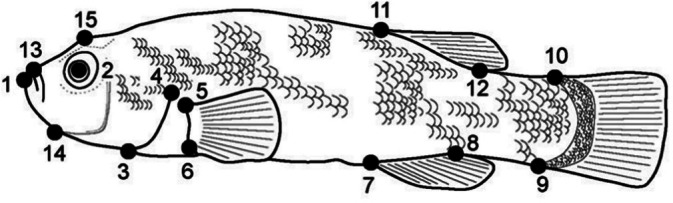
Illustration of *Orestias agassii* to indicate the landmarks (black points) from anatomically homologous point utilised: (1) beginning of mandible, (2) centre of eye, (3) base of isthmus, (4) beginning of gill cover or operculum, (5) and (6) insertion of pectoral fin, (7) and (8) beginning and end of anal fin, (9) and (10) insertion of caudal fin, (11) and (12) beginning and end of dorsal fin, (13) beginning of pre‐maxilla, (14) sub‐mandibular union, (15) dorsum of head (From Cruz‐Jofré et al., 2014).

We captured images of individual *Orestias* from the left lateral view using a digital camera (Canon EOS 450 D). We used tpsDIGw32 (Rohlf, 2015) to digitise 15 landmarks from anatomically homologous points following Cruz‐Jofré et al. (2014) (Figure 2). The landmark coordinates of the specimens were aligned using generalised Procustes superimposition. This orthogonal transformation allows landmarks to be standardised and scaled for comparison (Dryden & Mardia, 1998). This method aims to extract shape variation by eliminating the non‐shape components of variation, that is, size, position and orientation (Klingenberg, 2015).

### 2.6 Data analysis

#### 2.6.1 Stable isotope values

We visualised stable isotope values of *O. chungarensis* liver and muscle tissues and their putative prey using δ^15^N–δ^13^C, δ^15^N–δ^34^S and δ^1^ ^3^C–δ^34^S biplots. Then we examined possible diet shifts through tests of correlation (Kendall's test) between fish size (fork length) and stable isotope values (δ^13^C, δ^15^N and δ^34^S). Visual observations of stable isotope values indicated that an isotopic split was apparent in the data, with individual fish falling into two separate groups. Subsequently, a *k*‐means cluster analysis was performed using variables of δ^13^C, δ^15^N and δ^34^S isotope values of *O. chungarensis* liver and muscle. Different graphical methods (Elbow, Silhouette, Hubert and D index) were used to evaluate the most likely number of clusters estimated from the *k*‐means cluster analysis (Kodinariya & Makwana, 2013). We then used discriminant function analysis to examine the reliability of fish assigned into two clusters: *Orestias* A (*n* = 31, 74 %) and *Orestias* B (*n* = 11, 26 %). δ^13^C, δ^15^N and δ^34^S isotope values of *O. chungarensis* liver and muscle were used as a predictor.

#### 2.6.2 Diet: Stomachs contents

To examine the diet *of O. chungarensis*, we calculated mean %Fi and %Pi based on seven categories. We then arcsine‐square root transformed the %Pi data from each individual (Quinn & Keough, 2002) and generated a Bray–Curtis dissimilarity matrix in the vegan package (Oksanen et al., 2022) for R (version 4.3.2; R Core Team, 2023) using the R Studio IDE (version 2023.10.31; RStudio Team, 2023). We used the vegan function *adonis2* to conduct a permutation‐based (*n* = 9999) multiple analysis of variance (Anderson, 2001) to compare the diet of *O. chungarensis* between the two groups: *Orestias* A and *Orestias* B.

#### 2.6.3 Stable isotope mixing models

We estimated the relative contribution of different putative prey to the assimilated diets of *Orestias* A and *Orestias* B over time using the Bayesian mixing model *simmr* in R (Parnell, 2016). We used mean ± SD trophic discrimination factors for C and N in liver and muscle tissues from Canseco et al. (2021): δ^13^C_liver_ (0.6 ± 1.3 ‰), δ^15^N_liver_ (2.8 ± 1.6 ‰), δ^13^C_muscle_ (1.7 ± 2.0 ‰) and δ^15^N_muscle_ (3.7 ± 3.2 ‰). For S, we used mean ± SD values from an unpublished meta‐analysis (Harrod, unpublished): δ^34^S_liver_ (1.2 ± 0.1‰) and δ^34^S_muscle_ (1.3 ± 1.3 ‰). Putative prey were selected reflecting their contribution to stomach contents (Table 2) and from previous *Orestias* diet studies (Guerrero et al., 2015). Models were run using six distinct sources (pelagic zooplankton and amphipods; littoral amphipods, zooplankton and chironomids). To allow a more generalised indication of *O. chungarensis* consumption patterns by habitat, we then combined posterior estimates of relative contribution into groups (pelagic, littoral and sublittoral) using the *combine_sources*() function in *simmr*.

#### 2.6.4 Isotopic niche volume

We used the R package *nicheROVER* (Swanson et al., 2015) to calculate three‐dimensional isotopic niche volume for *Orestias* A and *Orestias* B. The isotopic niche was defined as a three‐dimensional volume (‰^3^) with a 95% probability of finding a specific sample from the group of interest, that is, reflecting short‐ (liver) or long‐term (muscle) isotopic values, and was denoted as NR_95_. Posterior distributions were obtained for each group (10,000 runs), and the niche size and overlap estimated. Levels of overlap were assessed by comparing posterior means of niche overlap with 95% credible intervals.

### 2.7 Shape analysis

We performed a principal component analysis (PCA), a well‐established, routine approach to visualise the general patterns of morphological variation. This analysis was used to reduce the set of variables to a small number of interpretable axes (PCs) (Zelditch et al., 2004). PCA was performed using the shape coordinates to identify the main axes of body shape variation between *Orestias* groups. Warp transformation grids were used to aid visualisation. A discriminant function analysis was run to examine the separation between *Orestias* groups. All analyses were performed in MorphoJ software (version 1.07a) (Klingenberg, 2011).

## 3 RESULTS

The mean ± SD length and mass of *O. chungarensis* were 72 ± 10 mm and 5.3 ± 2.9 g, respectively. Due to an unbalanced sex ratio in the survey catch (45 females: 1 male: 3 indeterminate), all data relate to female fish.

### 3.1 Stable isotope values

**FIGURE 3:**
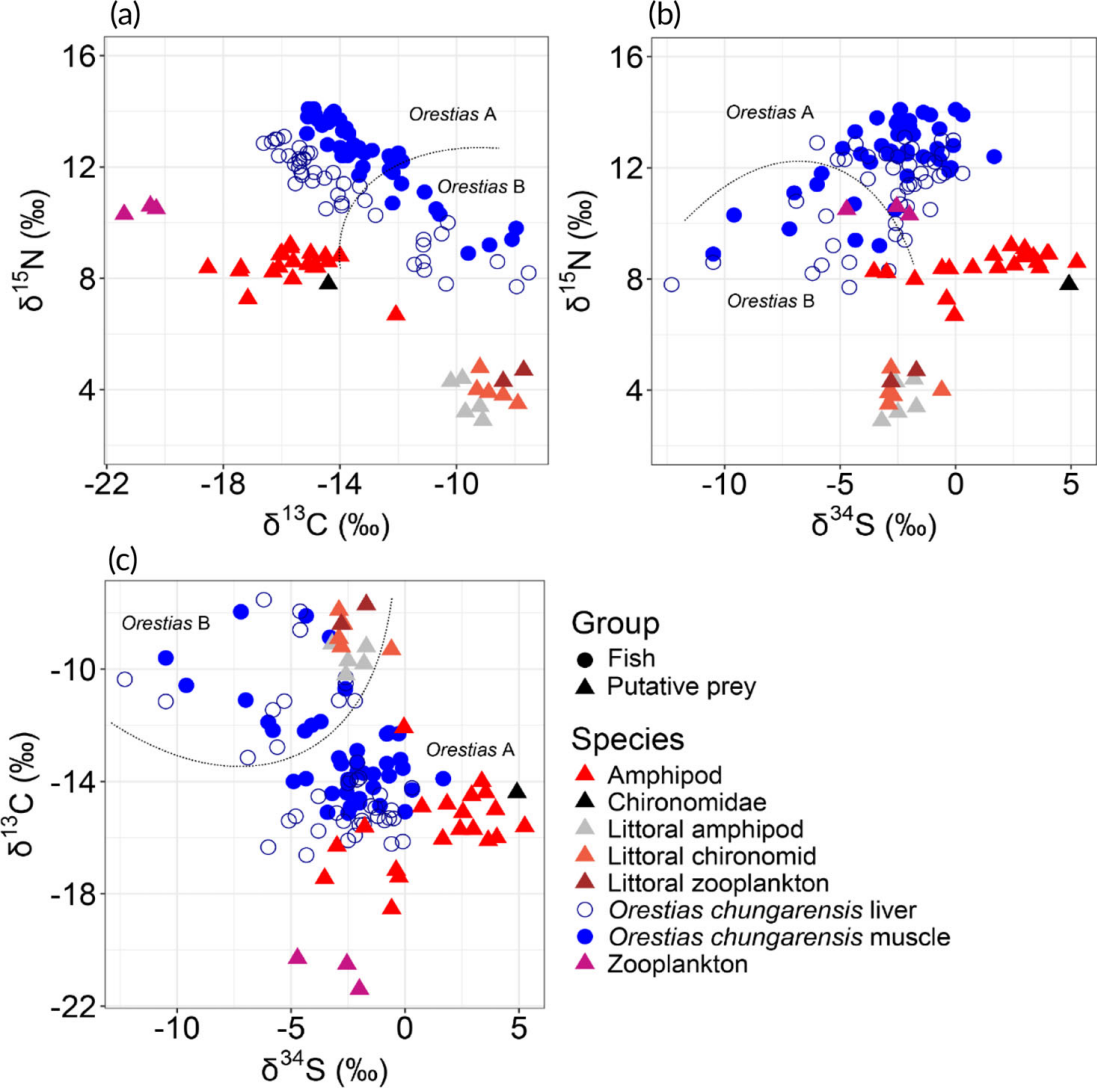
Stable isotope (a) δ^15^N–δ^13^C, (b) δ^15^N–δ^34^S and (c) δ^13^C–δ^34^S biplots showing individual values for *O. chungarensis* in liver (dark blue open circles), muscle (blue filled circles) and putative prey (filled triangles). δ^13^C values for *O. chungarensis* (but not their prey) have been corrected for lipid effects. The dotted lines indicate the separation between the two groups of *Orestias* identified by *k*‐means clustering.

Isotopic composition (δ^13^C, δ^15^N and δ^34^S) measured in liver and muscle of *O. chungarensis* and putative prey is shown in Figure 2. Values of δ^13^C, δ^15^N and δ^34^S from liver (a short‐term indicator: ~ 6 weeks) and muscle (a long‐term indicator: ~ 6 months) showed broad ranges given that they were from individuals of a single putative population (Figure 3; Table 1).

**TABLE 1:** Summary statistics [mean, standard deviation (SD), minimum and maximum values] of liver and muscle isotopic values of *O. chungarensis*.

Isotope	Tissue	Mean (‰)	± SD (‰)	Minimum (‰)	Maximum (‰)
δ^13^C	Liver	−13.7	2.4	−16.6	−7.5
	Muscle	−12.9	1.8	−15.1	−8.0
δ^15^N	Liver	11.1	1.6	7.7	13.1
	Muscle	12.3	1.4	8.9	14.1
δ^34^S	Liver	−3.2	2.6	−12.3	0.3
	Muscle	−3.0	2.7	−10.5	1.7

**FIGURE 4:**
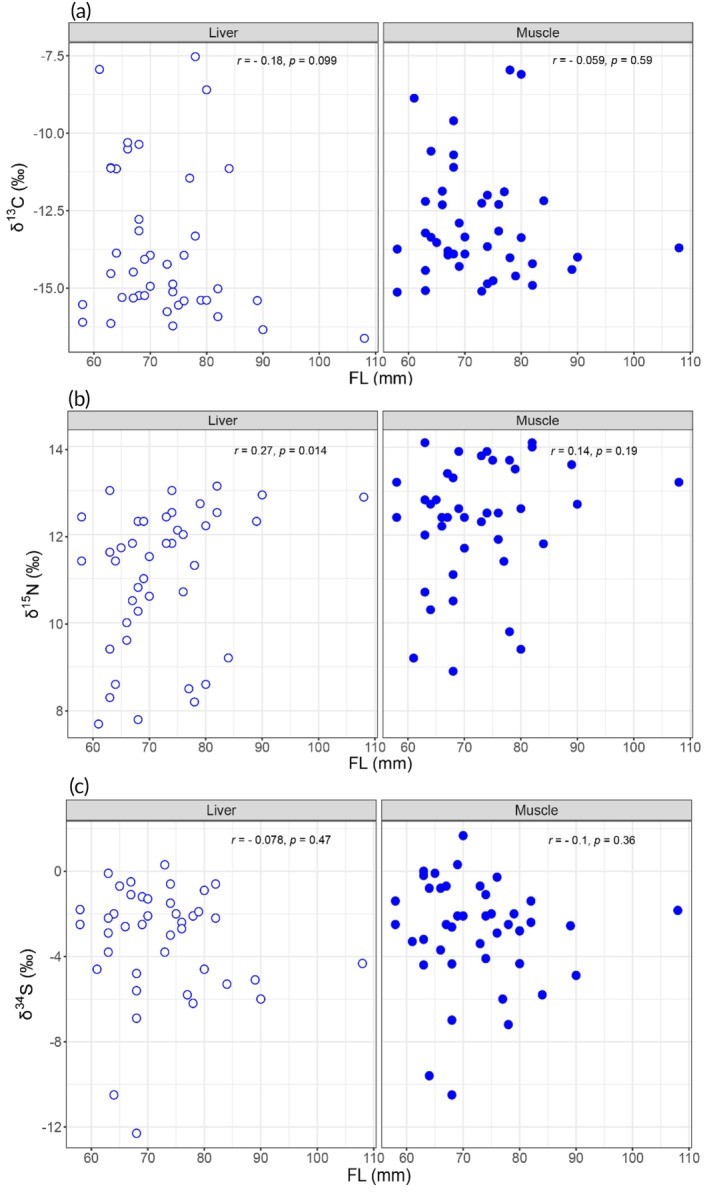
Scatterplots showing variation in (a) δ^13^C, (b) δ^15^N and (c) δ^34^S values from liver and muscle tissue as a function of fork length of *O. chungarensis*.

Correlation analyses examining possible relationships between *Orestias* size (FL) and stable isotope values (δ^13^C, δ^15^N and δ^34^S) of liver and muscle indicated that inter‐individual isotopic variation was not correlated with individual size (Figure 4). *Orestias* showed a weak positive correlation (*r* = 0.27, *p* = 0.01) between FL and liver δ^15^N values.

### 3.2 Cluster analysis

*K*‐means cluster analysis results using δ^13^C, δ^15^N and δ^34^S liver and muscle tissue isotopic values of *O. chungarensis* showed that *O. chungarensis* individuals were best separated into two groups: *Orestias* A with ^13^C‐depleted (relatively low δ^13^C), ^15^N‐enriched (relatively high δ^15^N) and ^34^S‐enriched (relatively high δ^34^S) values, and *Orestias* B with ^13^C‐enriched, ^15^N‐depleted and ^34^S‐depleted values (Figure 3; Table 2).

**TABLE 2:** Summary statistics [mean, standard deviation (SD)] of liver and muscle isotopic values of *O. chungarensis* groups: *Orestias* A and *Orestias* B identified by *k*‐means clustering.

Group	*n*	Tissue	δ^13^C (‰)	δ^15^N (‰)	δ^34^S (‰)
*Orestias* A	31	Liver	−14.9 ± 1.4	11.8 ± 0.9	−2.2 ± 1.5
		Muscle	−13.8 ± 0.9	13.0 ± 0.7	−1.8 ± 1.5
*Orestias* B	11	Liver	−10.5 ± 1.8	8.9 ± 1.0	−6.1 ± 2.9
		Muscle	−10.5 ± 1.6	10.5 ± 1.1	−5.9 ± 2.5

### 3.3 LDA analysis

**FIGURE 5:**
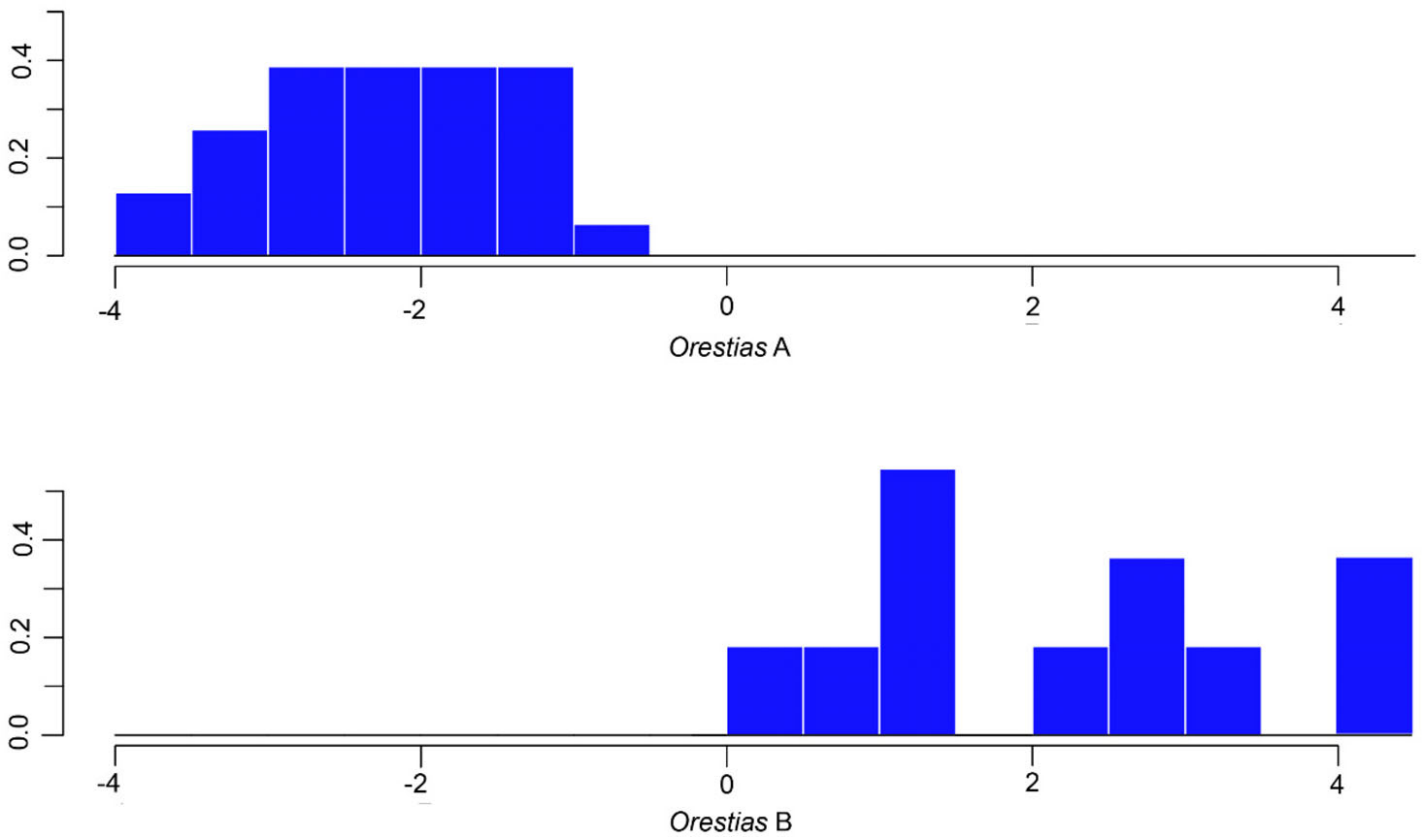
LDA plot of mean scores of discriminant function showing the two groups of *O. chungarensis*: *Orestias* A and *Orestias* B.

**FIGURE 6:**
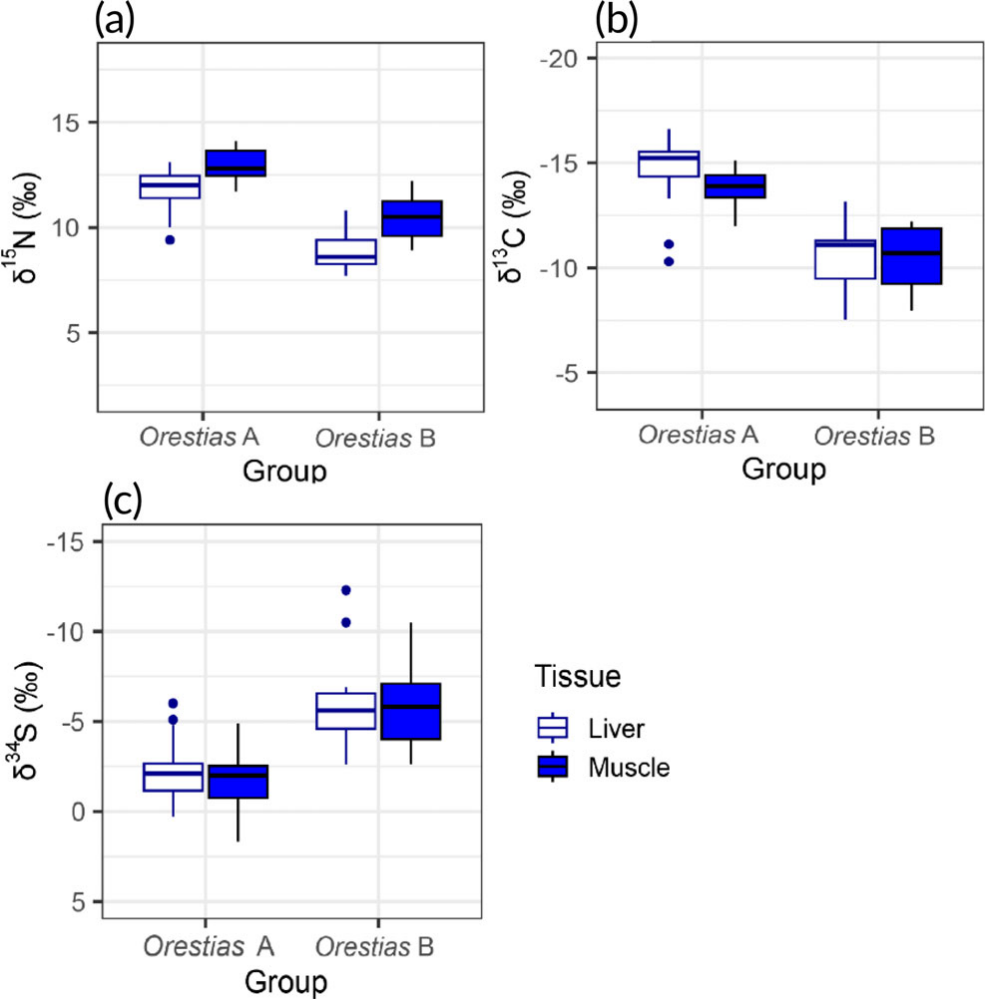
Boxplots comparing the distribution of nitrogen, carbon and sulphur stable isotope values from liver (dark blue) and muscle (blue) tissues of *Orestias* A and *Orestias* B captured from Lake Chungará's catchment. Solid horizontal lines represent the median values; boxes represent the interquartile range; and whiskers represent the variability beyond the interquartile range (IQR) (i.e., Q1–1.5*IQR, Q3 + 1.5*IQR).

A discriminant function analysis using δ^13^C, δ^15^N and δ^34^S liver and muscle tissue isotopic values as predictors supports the existence of two distinct *Orestias* groups (Pillai's trace test = 0.8, approximately F_6,35_ = 22.85, *p* < 0.001), with a jack‐knifed cross‐validated classification success of 98%. The LDA plot of mean scores of discriminant function showed that *Orestias* had two distinct isotopic groups (Figure 5). The *Orestias* A group was positioned to the left (−) side of the LDA, and individuals were characterised by ^13^C‐depleted, ^15^N‐enriched and ^34^S‐enriched values. *Orestias* B individuals were positioned on the right (+) side of the LDA and were characterised by ^13^C‐enriched, ^15^N‐depleted and ^34^S‐depleted values (Table 2). Figure 6 shows the distribution of nitrogen, carbon and sulphur stable isotope values from liver and muscle tissues of *Orestias* A and *Orestias* B.

### 3.4 Diet: Stomach contents

Only a single *O. chungarensis* had an empty stomach. Stomach contents were statistically similar in fish identified isotopically as *Orestias* A and *Orestias* B [permutational multivariate analysis of variance (PERMANOVA): F_(1,41)_ = 1.33; *p* = 0.2]. Both measures of diet (frequency of occurrence and proportional contribution of prey) indicated that the two groups largely fed on amphipods, chironomid larvae/pupae and gastropods (Table 3).

**TABLE 3:** Summary statistics for stomach contents of *Orestias* A and *Orestias* B groups from Lake Chungará.

Group	*Orestias* A		*Orestias* B	
*N*	31		11	
Size range (mm)	58–108		61–82	
Taxon	%Fi	%Pi	%Fi	%Pi
Amphipoda (*Hyallela* sp.)	100	87	100	81
Gastropods (*Biomphalaria* sp. and *Ancyllus* sp.)	19	4	0	0
Chironomidae (pupae and larvae)	10	3	20	7
Digested material	3	2	10	8
Zooplankton (copepods)	0	0	10	1
Unidentified invertebrates	6	0.3	0	1

*Note*: Data are provided as mean percentage frequency of occurrence (%Fi) and contribution (%Pi).

### 3.5 Stable isotope mixing models

**FIGURE 7:**
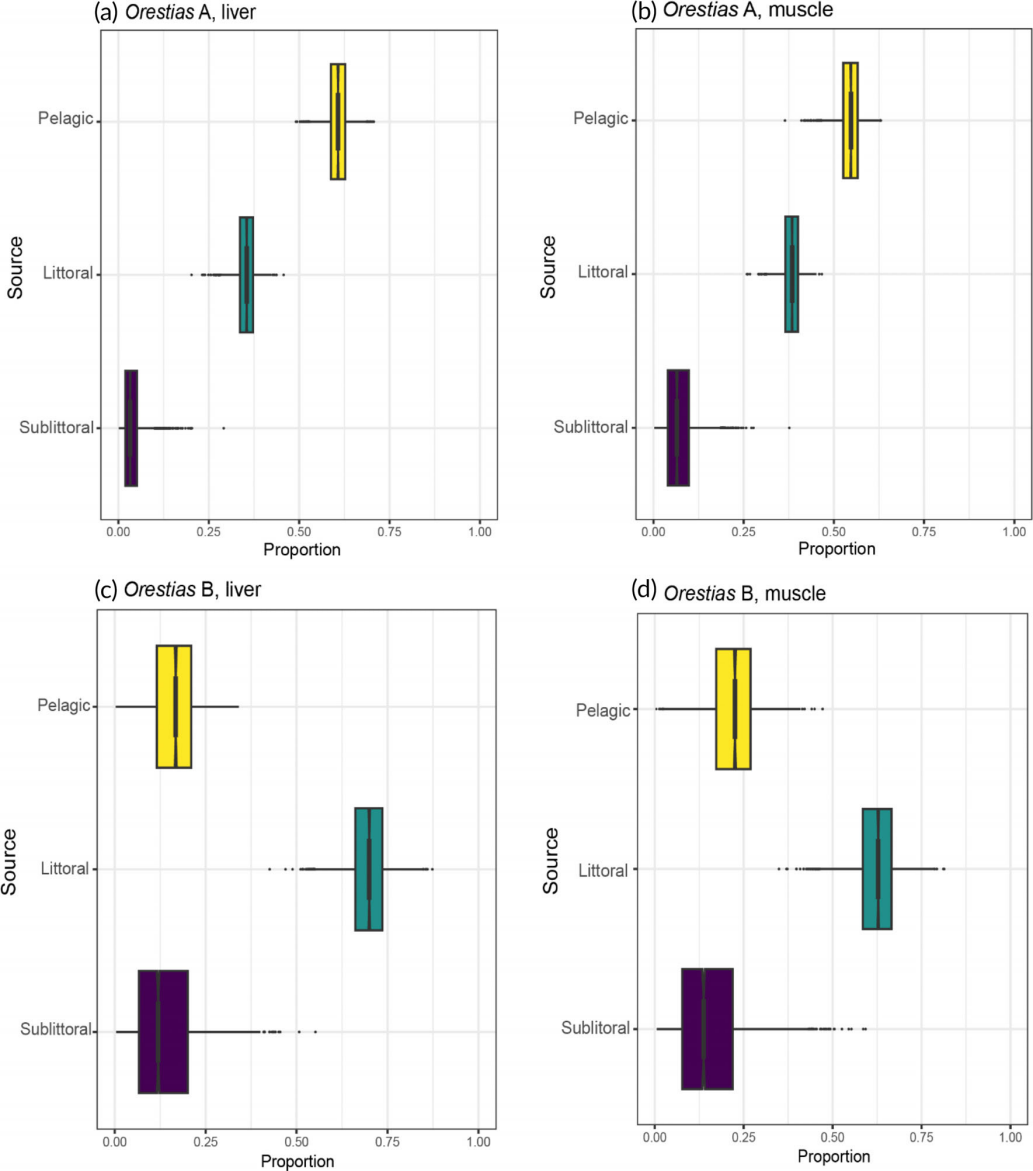
Visual representation shows simmr mixing model results by *Orestias* trophic group and tissue type. Boxplots showing the distribution of posterior estimates of the contribution of different putative prey to assimilated diet of *O. chungarensis*: (a) *Orestias* A, liver; (b) *Orestias* A, muscle; (c) *Orestias* B, liver; and (d) *Orestias* B, muscle.

Mixing model results showed that the percentage of contribution of different prey to assimilated diet of *Orestias* A and *Orestias* B was different and consistent over time (Table 4; Figure 7). Based on liver tissues (a relatively short‐term indicator), *Orestias* A assimilated much of their diet from both pelagic (median contribution = 61%) prey and littoral prey (36 %). Contributions from sublittoral prey were low (<5 %). Muscle tissues (a long‐term indicator) indicated a similar contribution of pelagic prey (55 %) and a slight increase in littoral prey contribution (38 %). Conversely, a similar analysis of *Orestias* B assimilated diet indicated that these fish consumed largely littoral prey over both the short term (liver: 70 % littoral) and long term (muscle: 63 %).

**TABLE 4:** Summary statistics [median (95% credibility limits)] for the estimated percentage contributions of different putative prey to the assimilated diet of *O. chungarensis* (liver) and long‐time (muscle) in different groups.

Group	*Orestias* A		*Orestias* B	
Tissue/Putative prey	Liver	Muscle	Liver	Muscle
Pelagic	61 (55–67)	55 (48–60)	21 (0.4–28)	15 (3–39)
Littoral	36 (30–40)	38 (32–43)	70 (58–79)	63 (50–73)
Sublittoral	3 (0.6–11)	6 (0.1–18)	12 (0.2–35)	14 (2–40)

### 3.6 Isotopic niche volume

Isotopic niche volume (95 %) estimated using δ^13^C, δ^15^N and δ^34^S values showed that *Orestias* A had a smaller isotopic niche than *Orestias* B based on values estimated from liver [mean ± standard error (SE) liver isotopic niche volume: *Orestias* A: 78.4 ± 17.5 ‰^3^; *Orestias* B: 239.6 ± 95.8 ‰^3^] and muscle tissue (*Orestias* A: 49.1 ± 11.1‰^3^; *Orestias* B: 174.8 ± 69.2 ‰^3^). Comparisons of isotopic niche size from liver and muscle tissue within each group indicated that muscle tissues had a smaller isotopic niche size compared to liver tissues.

Comparisons of isotopic niche measured using isotopic values of liver tissues (reflecting short‐term diet) of *Orestias* A and *Orestias* B individuals showed an average overlap of 17.6 %. Isotopic niche overlap increased to 33 % using muscle tissues (longer‐term diet). Comparisons of isotopic niche overlap within each group, that is, between liver and muscle tissues showed considerable differences: *Orestias* A showed an inter‐tissue overlap of 8.3 % and *Orestias* B of 24 %.

### 3.7 Shape analysis

**FIGURE 8:**
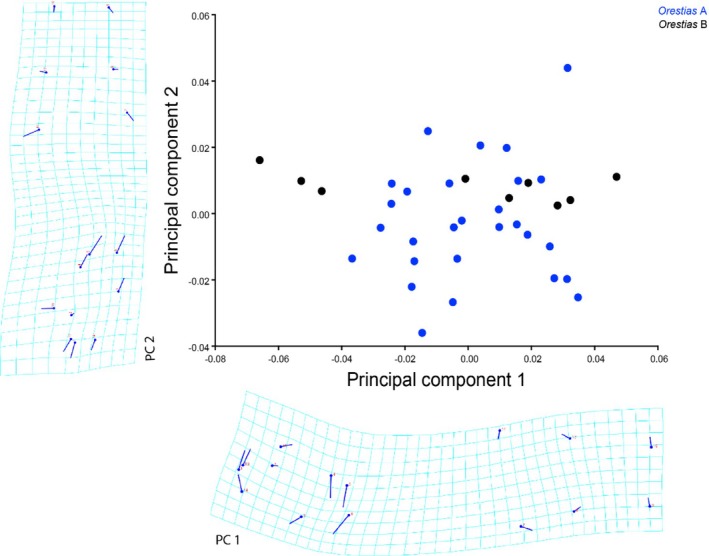
Graph shows a principal component analysis (PCA) using the shape co‐ordinates as a component (PC 2) of *O. chungarensis* groups.

The morphology of both putative *O. chungarensis* groups was explored using PCA ordination and deformation grids to visualise shape variation (Figure 8). Visual assessment of the PCA ordination and deformation grids showed no strong differences in shape between the groups. The results of the discriminant function analysis were on the threshold of significance (T‐ square = 236.5, *p* = 0.06), underlining the lack of marked differences in the body shape of *Orestias* A and *Orestias* B.

## 4 DISCUSSION

To date, studies of the ecology of *O. chungarensis* have only included their initial description (Vila & Pinto, 1986) and a characterisation of their diet (Guerrero et al., 2015). Both studies relied on individuals captured from shallow littoral habitats, and Guerrero et al. (2015) only based their studies of *O. chungarensis* diet on stomach contents. Our study provides the first trophic characterisation of *O. chungarensis* from individuals captured in different lake habitats (depths) and using multiple approaches. This approach has provided a deeper understanding of resource use and foraging habitat use in the endemic *O. chungarensis*.

Our results demonstrated the existence of marked intrapopulation variation in the isotopic (δ^13^C, δ^15^N and δ^34^S) composition of *O. chungarensis* in tissues indicative of both shorter (liver) and longer (muscle) assimilation periods. Stable isotope values provide an individual‐level record of trophic ecology and habitat use (Harrod et al., 2005, 2010). As stomach contents were similar across the individuals examined here, the existence of isotopic differences in both liver (short‐term) and muscle (long‐term) tissues indicates that the two groups forage in habitats that differ in isotope composition. Given evidence from other systems (Harrod et al., 2010; Hayden et al., 2014; Vander Zanden & Rasmussen, 1999), it is likely that such isotopic variation is driven by foraging at different depths (or distance from shore) where biochemical differences associated with lake bathymetry are transferred to the food web.

Within‐species variation in diet and habitat use reported from other *Orestias* species has largely been associated with fish size (e.g., ontogenetic dietary shifts) and sex (Guzmán & Sielfeld, 2009; Pinto & Vila, 1987; Riveros et al., 2012, Flores‐Arzabe, 2013). However, the isotopic variation seen here in *O. chungarensis* was from individuals of similar size, shape, sex (we only considered adult females in this study) and diet. Our analysis of the broad ranges of isotopic values showed by *O. chungarensis* in Lake Chungará indicates that the lake supports at least two trophic groups. Fish from these two groups consume broadly similar prey but were isotopically distinct, which, we suggest, reflects feeding in spatially distinct habitats. Our results reveal that the bulk of the sample captured (*n* = 31, 74%) was associated with deeper offshore habitats (*Orestias* A: ^13^C‐depleted, ^15^N‐enriched and ^34^S‐enriched), whereas a smaller proportion of individuals (*n* = 11, 26%) were associated with shallower littoral habitats (*Orestias* B: ^13^C‐enriched, ^15^N‐depleted and ^34^S‐depleted). The results of stable isotope mixing models showed differences in assimilated diet proportion sources between the two groups. *Orestias* A assimilated more energy and nutrients from prey (zooplankton) with pelagic‐derived stable isotope values. *Orestias* B were more associated with prey (amphipods, chironomids and zooplankton) with littoral‐derived values.

Few stable isotope data have been published from other *Orestias* species. *O. chungarensis* from Lake Chungará were both ^13^C‐ and ^15^N‐enriched relative to those from Lake Titicaca, reflecting limnological differences between the two lake systems. However, the wide scale of isotopic variation (range: δ^13^C = −15.1 to −8.0 ‰; δ^15^N = 8.9–14.1‰) shown by *O. chungarensis* in Lake Chungará is comparable to that of the combined isotopic variation (range: δ^13^C = −20.7 to −14.3 ‰; δ^15^N = 5.0–11.5 ‰) shown by all 14 distinct *Orestias* species from Lake Titicaca (Monroy et al., 2014). The level of isotopic variation shown by *O. chungarensis* in Lake Chungará provides strong evidence that its trophic ecology is more complicated than has been assumed to date, especially given that isotopic values varied markedly in both liver and muscle tissues.

Since its formation, about 10,000 years ago, it is highly probable that *O. chungarensis* was the only fish species inhabiting Lake Chungará until the recent arrival of rainbow trout. Following patterns typical of radiations seen in fishes from depauperate lakes (Skúlason et al., 2019), *O. chungarensis* may have occupied niches associated with benthic, pelagic and littoral habitats (Vila et al., 2007), as seen in other *Orestias* sp. in the *agassii* complex (Parenti, 1984a). Given the short generation time of *Orestias* spp. (1–2 years), and the existence of multiple vacant niches, this indicates ample time and opportunity for ecological divergence into at least two ecotypes of *O. chungarensis*. This is reflected in our results that show that the two *Orestias* groups have distinct isotopic niche volumes and little (17% – 33%) isotopic niche overlap, which, we suggest, provides further evidence of divergence in habitat use.

Ecological divergence based on habitat segregation has been repeatedly reported from depauperate lake fish communities (Robinson & Wilson, 1994; Skúlason & Smith, 1995; Taylor, 1999). Other *Orestias* species within the *agassii* complex show considerable morphological variability that is associated with occupation of specific microhabitats and different foraging modes (Maldonado et al., 2009; Flores‐Arzabe, 2013; Loayza et al., 2021). The apparent overlap in stomach contents and body shape shown between the isotopically distinct groups of *O. chungarensis* therefore indicates that the differences are likely subtle, and that the two groups feed similarly but in distinct habitats. Future studies need to examine whether there is any evidence for genetic differentiation between the two groups of *O. chungarensis* identified here.

## 5 CONCLUSIONS

Our study revealed that *O. chungarensis*, the sole endemic fish inhabiting Lake Chungará, displays more ecological diversity than previously considered (Guerrero et al., 2015; Vila et al., 2007; Vila & Pinto, 1986). Our use of SIA has revealed previously cryptic habitat‐structuring, where fish feed on similar prey (amphipods, gastropods, chironomids and zooplankton) but in distinct habitats (likely different depths). These findings highlight the plasticity of *Orestias* spp. and their capacity to adapt to habitat heterogeneity available in high‐altitude freshwater ecosystems. Future work is required to focus on how *Orestias* A and *Orestias* B are segregating habitats, and this should examine physiochemical and isotopic variation across lake habitats and depths and whether these two putative groups are genetically distinct.

## AUTHOR CONTRIBUTIONS

Karina González and Chris Harrod conceived and designed the study. Karina González, Chris Harrod and Daniel Gomez‐Uchida collected the samples. Karina González processed the samples. Karina González and Chris Harrod analysed the data. Karina González, Chris Harrod and Daniel Gomez‐Uchida led the writing, review and editing of the manuscript.

## FUNDING INFORMATION

Millennium Nucleus of Austral Invasive Salmonids (INVASAL, Project NCN2021_056) funded by Chile's government programme ANID Millennium Science Initiative of the Ministerio de Ciencias, Tecnología, Conocimiento e Innovación (Chile). Karina González was supported by doctoral fellowships from ANID and the Universidad de Antofagasta (Beca de Excelencia Académica, Escuela de Postgrado).
